# Covid 19-the 21st Century Pandemic: The Novel Coronavirus Outbreak and the Treatment Strategies

**DOI:** 10.34172/apb.2022.005

**Published:** 2021-01-30

**Authors:** Sherin Joseph, Anila Kutty Narayanan

**Affiliations:** ^1^Department of Pharmacy Practice, Amrita School of Pharmacy, Kochi, Amrita Vishwa Vidyapeetham, India.; ^2^Faculty of Department of Pharmacy Practice, Amrita School of Pharmacy, Kochi, Amrita Vishwa Vidyapeetham, India.

**Keywords:** ACE2 receptors, Antiviral therapy, Coronavirus, COVID-19, Drug repurposing, SARS-CoV-2, Vaccines

## Abstract

COVID -19 a global pandemic that has brought all the greater global countries to a hook. The novel coronavirus (SARS-CoV-2) outbreak was first reported in Wuhan, China which then started spreading to different countries around the world. ACE2 receptors are present in various organs but the overexpression of ACE2 at lung epithelia makes them more vulnerable to respiratory symptoms. SARS-CoV-2 binds to ACE2 receptors for entry into host cells which may serve as potential target for future therapy.Repurposing of drugs are the present strategy undertaken as the SARS-CoV-2 shows similar respiratory distress symptoms as in the case of SARS and MERS. At present the antiviral medications and vaccines are at the early stages and may take few months to years, to achieve their complete efficacy to solve the public crisis. The technological advancements have brought passive immunisation, which is an anecdotal success, but the ideal approach to future outbreaks of SARS-CoV-2 is done by vaccines that are under clinical trials. There are a large percentage of population under psychological crisis either due to the fear of infection or stress from the quarantine lives. High levels of viral loads at the initial stages cause higher chances of transmission hence immediate isolations and screening methods must be undertaken. This review mainly focuses on the treatment strategies followed with no definitive approval from authorities. This is an attempt to gather all the materialistic evidences available for now.

## Introduction

The emergence of novel coronavirus disease 2019 (COVID-19) is the latest pandemic that is affecting human health and economy across the world.^1^ A novel strain of coronavirus (2019-CoV) was detected in Wuhan, Hubei province, China in December 2019. Coronavirus infects both humans and animals including severe acute respiratory syndrome coronavirus 2 (SARS-CoV-2) in Southern China 2003 and Middle East respiratory syndrome–related coronavirus (MERS-CoV) in Saudi Arabia 2012 were two severe forms of infections out broken in the past.^2,3^ There are six coronavirus species well known to affect human, of which one species subdivided into two different strains making seven strains of human coronavirus. Out of this four strains (HCoV-HKU1, HCoV-229E, HCoV-OC43, HCoV-NL63) mostly cause common cold symptoms and three strains (SARS-CoV, MERS-CoV, SARS-CoV-2) cause severe acute respiratory syndromes. SARS-CoV and MERS-CoV can cause fatal respiratory diseases. As of now the magnitude of the outbreak exceeds the potentially available medical management capacities which make the scenario serious. Hence, the Nations worldwide are trying to control this havoc. The novel coronavirus will be referred as SARS-Cov-2, and has been labelled as a public health emergency of international concern as the pandemic curves are still on the rise.^3,4^ The coronavirus pandemic has led to severe global outbreak leading to socioeconomic disruption and has hence become a major public health issue. The infection has estimated to have mean incubation period of 5-6 days and a basic reproduction number of 2.24-3.58. COVID-19 had a case fatality rate ratio of 4% however recent reports suggests between 1 to 2 %.^5^ The novel coronavirus infection is called a super spreader as it can be spread from an individual patient to a large population of different countries which led the WHO characterise COVID-2019 as a pandemic crisis. The epidemiological studies suggest, the COVID -19 is known to be mostly fatal for elderly patients with comorbid conditions. The coronavirus are divided into four genera-alpha, beta, gamma and delta.SARS-CoV-2 belong to gene betacoronavirus^6^ wherein phylogenetic analysis revealed that the major reservoir of CoV infections are bats and bat-SL-CoVZC45, bat-SL-CoVZXC21 are two bat derived SARS like coronavirus which shows 79% similarity to SARS and 50% similarity to MERS.^3,7^ As there are many clinical trials undertaken by various countries to isolate and identify drugs and vaccines for COVID-19 some of the prevailing treatment strategies are discussed further which is not so far a standard protocol.

## Epidemiology

On 29^th^ December 2019, the first case of COVID-19 was reported in the Hubei province, Wuhan city, China. Four people from the street seafood market were admitted in the hospital and showed respiratory symptoms with fever.^3^ As a second outbreak, there were more human to human transmissions at a higher rate suggesting it to be an epidemic and viral infection. The cases reported included both immunosuppressed and normal people, with majority belonging to middle age and older citizens.^8^

## Coronavirus

Coronaviruses are enveloped non-segmented single stranded positive sense RNA virus with a large size genome belonging to the family Coronaviridae, order Nidovirales and broadly infects humans and other animals.^7,9^ The inclusion criteria also explains that the patients of age greater than 60 are at greater mortality risk therefore initiation of combination therapy can be preferred. The structure of the virus is spherical virion surrounded with projections, hence corona in Latin means crown. The genome size varies from 26 kb to 32 kb. The SARS-CoV -2 belongs to B-lineage of beta coronavirus. There are four major genes that encode the nucleocapsid-the spike protein (S), a membrane glycoprotein (M), a small membrane protein (SM), an additional membrane glycoprotein (HE).^6,10^ The reproduction number of COVID-19 is greater than SARS 1.77. The incubation period are expected to be an average of 7-14 days. The coronavirus exhibits zoonotic nature that includes respiratory, enteric, hepatic and neurological problems. The transmission is basically by droplet route, direct contact and aerosols route. As the patients diagnosed with SARS-CoV was found to have abdominal discomfort and diarrheal symptoms, the analysis opens a direct relation between angiotensin-converting enzyme 2 (ACE-2)^11^ receptors and coronavirus transmission. The SARS–CoV-2 encodes several proteins including structural proteins, on-structural proteins and auxiliary proteins. The spikes present on the external side of the virus binds to the ACE2 receptor.^12^ They contain amino acids with N- terminal S1 and C-terminal subunits. The S1 subunit has receptor binding domain (RBD) containing two subdomains. Out of it the external subdomain has two loops on the surface that binds to ACE2 receptor. This may explain the reason of entry of SARS-CovV-2 through receptors like ACE2 in respiratory tract.^11,13^ The ACE-2 receptors are present on small and large arteries, nasal-oral mucosa, nasopharynx, enterocytes of the small intestine but being abundant on the lung epithelia, pulmonary symptoms dominate in COVID-19 infections.^14^ Therefore, targeting the ACE-2 receptors, pneumonia-like symptoms along with pulmonary oedema and ARDS can be reduced.^15^ Coronavirus spike (S) protein is the major site responsible for viral attachment to the host ACE-2 receptors thus drugs carried by appropriate vector systems targeting viral S proteins can diminish the viral load at the target site.^16^

## Symptoms

The most common symptoms of COVID-19 at onset are fever (90%), dyspnea, fatigue (70%), dry cough (60%), myalgia (44%) and less common symptoms are headache, dizziness, diarrhoea, nausea and vomiting. The elderly patients having underlying co-morbidities including hypertension, diabetes, and cardiovascular disease are at greater risk. The most common laboratory abnormalities among patients hospitalised with COVID-19 are marked lymphopenia, prolonged prothrombin time, elevated LDH, elevated D-dimer and these are close to the ones in MERS-CoV and SARS-CoV infections.^2,3,17^

The studies provide evidence that severe COVID-19 cases might have a cytokine storm syndrome. The cytokine storm is associated with the increase in cytokines like IL-2, IL-7 granulocyte colony stimulating factor, interferon-gamma, macrophage inflammatory protein 1-alfa and tumour necrosis factor-alfa. Viral infections and sepsis may trigger the secondary haemophagocytic lymphohistiocytosis (sHLH), which is characterised by unremitting fever, cytopenia’s, hyperferritinaemias, and acute respiratory distress syndrome (ARDS).

## Histological examinations

The dysregulated and exuberant immune responses due to coronavirus infections cause alveolar damage and respiratory failure leading to diminished survival rates. As cytokines and chemokines have a role in immunity and immunopathology the increase in cytokine levels (interleukin-6, 10 and tissue necrosis factor-alfa) with reduction in IFN-gamma expression of CD4 T-cells are considered as indicators of severe COVID-19.^2^ Histological examination usually shows bilateral alveolar damage with cellular fibromyxoid exudates, shedding of pneumocytes and hyaline membrane formation indicating ARDS,^18^ pulmonary oedema, moderate microvesicular steatosis, levels of CD4 and CD8 were substantially reduced and high concentration of proinflammatory (CCR6 + Th17) CD4 T-cells showing viral cytopathic changes. Lymphopaenia is a critical factor associated with disease severity and mortality of COVID-19 patients. The COVID-19 infection primarily targets respiratory system causing severe pneumonia, and the epithelial cells of lungs are damaged.^19^

## Pathological evidence

The pulmonary pathological features of SARS-CoV from a post-mortem examination done in China explain pleural effusion causing pleural oedema and extensive consolidation. Majority of patients had diffuse alveolar damage (DAD), and hyaline membrane formation.^20^ There were some cases of interstitial thickening, moderate fibrosis, and presence of inflammatory cells. Dilation of airspaces and intra alveolar organisations of exudates, granulation of tissues in airways and airspaces, lesions of cellular components of histiocytes. The presence of large multinucleated pneumocytes were found from most post-mortem. The lymphoid infiltrate consisted of sparse T-cells and few B-cells but natural killer cells were absent.^19^ Most of the cells with atypical nuclei were positive for cytokeratin indicating the presence of pneumocytes. The viral like particles on pneumocytes depicts the presence of nucleocapsids and the surface of some had club like projections. There was also a ring moiety in some particles indicating the characteristics of helical nucleocapsid of coronaviruses. The pulmonary fibrosis found in patients largely points to the SARS infection. These interventions suggest that pneumocytes are the primary target for SARS-CoV infections.^13^ The pathogenesis involves proteolytic cleavage and membrane fusion mechanisms that serve their entry and spread. The virus once entered sheds its viral RNA genome in the cytoplasm that undergoes replication by post-translational events to produce viral glycoproteins. These get assembled in the endoplasmic reticulum and Golgi complex that enables the formation of vesicles of nucleocapsid proteins that fuse with the host plasma membrane to release new virions.^21^

Recent literatures have shown various extrapulmonary systemic involvement of disease articulation and virus transmission which included gastrointestinal tract, nervous system, cardiovascular system, renal system, eyes, and manifestations due to haematological abnormalities. Post COVID-19 complications include enduring damage to the heart muscle, even in those with only mild symptoms, increasing the risk of heart failure or other heart complications. Strokes, seizures, and Guillain-Barre syndrome that causes temporary paralysis. Possibilities of developing Parkinson’s and Alzheimer’s disease. Problems associated with blood cells like clotting leading do heart attacks, stroke has been studied. Heart damage caused by COVID-19 is believed to occur from exceedingly small clots that block capillaries in the heart muscle. Renal disorders, hypertension, hyperglycaemia, mood disorders, septic shock, extreme fatigue, and myalgia have been reported recently. Multisystem inflammatory syndrome is also said to occur in both elderly and paediatric groups.^22^

## Incubation period

The epidemiological key parameter of COVID-19 is the incubation period which is determined with the help of epidemiological cases reporting. Incubation period of an infection is necessary factor to identify the quarantine periods. This also helps in identifying the contact tracings and screening purposes and transmission potential strength. Now the incubation periods are estimated with the data available from the reported travel histories and symptom onset dates. Accordingly, the mean incubation period ranges from 1-14 days with a median of 5-7 days, which may be chosen for the quarantine periods.^23^

## Reproduction number (Ro)

The studies using stochastic method helped to estimate Ro of SARS to lie within 2 and 5 which is within the range of the mean Ro of COVID-19 also suggesting the reproduction number Ro of COVID-19 is greater than SARS coronavirus. The reproduction number helps to identify the transmission level and the incidence of new infections. The intervention that COVID-19 is more widespread than SARS makes it more transmissible. The review found that the mean average of Ro in case of COVID-19 is 3.28 with median of 2.79, and IQR of 1.16.^24^

## General treatment approaches

Currently there are no definitive proven treatments, thus multiple pharmacological options are being explored. The novel coronavirus prefers the ACE2 receptor for penetrating into the cells, therefore strategies used for blocking SARS are being tried out. Some of the trials initiated by using the undermentioned drugs showed improvement in patients and some animal models that led to further interest.^7^ Treatment basically focus on the management of patients. Additional intensive care with broad spectrum antibiotics, glucocorticoids, vasopressor and renal replacement therapy may be undertaken during SARS-CoV-2 therapy. Drug therapy monitoring is crucial in case of antiviral therapies and require continuous health surveillance as they are more likely to cause metabolic and haematological abnormalities.^25^

Chloroquine, an antimalarial, has an in vitro activity against SARS-CoV-2 and may have immunomodulating properties. The drug mainly acts by increasing the endosomal pH that inhibits the fusion of viral particle and host cell membranes. Initially the pre-clinical data of in vitro studies suggests Chloroquine (CQ) had activity against SARS-CoV-2 and also reduce the exacerbations of pneumonia patients with COVID-19 infection.^10^ A systematic review conducted on safety and efficacy of chloroquine by Chinese researchers using Vero E6 cells infected by SARS-CoV-2 demonstrated that chloroquine was highly effective in reducing viral replication with an effective concentration (EC) 90 of 6.9 µM that can be easily achieved with standard dosing due to its favourable penetration into the tissues and lungs.^4^ Similarly the early stage pre-clinical in vitro data suggests that hydroxychloroquine with EC 50 values 0.72 µM may be more potent than chloroquine with EC 50 values 5.47 µM.^26^ An open non randomised clinical trial conducted showed (70%) of hydroxychloroquine treated patients were cured when compared to the untreated control group.^27^ In the United States, the FDA issued an emergency use authorization (EUA) to allow the use of these agents in adolescent or adults hospitalized for COVID-19 when participation in clinical trials is not feasible.^28^ Although the initial single centred clinical trials showed CQ/HCQ has therapeutic efficacy on COVID-19 patients the further follow up on larger sample size population has come to a contrasting conclusion. Usually there are mild side effects like dizziness, gastrointestinal reactions, vomiting, and diarrhoea while the drugs when taken for longer period depicts toxic effects like retinopathy, conduction disorders and cardiomyopathy. The removal of the drug from the COVID-19 treatment strategy is mainly due to the abnormality of cardiac conduction system that provides higher chances of fatal arrhythmias. As CQ/HCQ are narrow therapeutic index drugs acute CQ poisoning is associated with potential life-threatening cardiovascular disease which made the long-term use conclusion futile. Hence regular monitoring of cardiac functions is necessary to rule out potential cardiac toxicity. There is still a need for potent evidences from multicentre clinical trials to clarify the safety and efficacy of CQ/HCQ in COVID-19 treatment trends.^29^ Even though the early stage treatment of HCQ to prevent viral replication was a success to an extent the patients must be cautioned while taking concomitant other antiviral QT prolonging drugs like azithromycin to avoid the pronounced side effect. The use of HCQ in healthy individuals and for a short period of time reduces the chances of life-threatening events. Monitor the QTc level of >500 ms (milliseconds) or an increase of 60 ms as they require immediate discontinuation of therapy and proper education counselling.^30^ The simple Vero cell models proves CQ and HCQ with appreciable antiviral properties but the more complex non-human primate models have no significant therapeutic benefit in SARS-COV-2 infections.^31^

Lopinavir and ritonavir are two HIV protease inhibitors that suppress the Mpro enzyme required for the coronavirus replication. Lopinavir is metabolised by CYP 3A isoenzyme in the liver, hence lopinavir is used with ritonavir as it helps to reduce the dose of lopinavir and ritonavir inhibits CYP3Aisoenzyme. The early stage data’s suggested that the adverse clinical outcomes like death and ARDS were lower in treatment groups when compared to control groups.^12,28,32^ As COVID-19 is an emerging virus, an effective treatment has not been developed for the disease, the Indian Council of Medical Research (ICMR) has suggested off label emergency use of lopinavir/ritonavir combination for symptomatic COVID-19 patients.^9,10^ But according to recent sophisticated multi cantered clinical trial studies lopinavir/ritonavir combinations require several days to achieve the high concentration of drug therefore a loading dose is appropriate which may often show mild gastrointestinal disorders. The delayed treatment with standard doses of lopinavir/ritonavir are unlikely to have benefit for blockage of viral replication.^33^ The randomised controlled open labelled RECOVERY trials between March 19 and June 29, 2020 of lopinavir/ritonavir verses placebo trials at UK provided new results different from the previous findings of the drugs efficiency in COVID-19 patients. The limitations of the drug involve the uncertainty in the dose of lopinavir/ritonavir to achieve adequate SARS-COV-2 inhibitory concentration at lungs, their maximum effective concentration is limited probably due to their high protein binding >95%. Hence the monotherapy of lopinavir/ritonavir in patients with COVID-19 did not provide beneficiary reduction in hospital stay or mortality rates.^34^

Remdesivir (GS-5734) is an investigational nucleoside analogue, broad spectrum anti-viral with in vitro activity against SARS-CoV -2. It is a prodrug of remdesivir triphosphate that acts as an inhibitor of RNA-dependent RNA polymerase (RdRp) and interrupts proof reading causing a decrease viral RNA production. Remdesivir – triphosphate competes with adenosine-triphosphate for incorporation into nascent viral RNA chain, once incorporated this, then terminates RNA synthesis at position (i+3) and cause chain termination. The vitro studies conducted using Remdesivir is on the clinical phase, evaluating the efficacy and safety of the drug in 452 hospitalized adult patients with severe COVID-19 respiratory disease.^7,12,35^ Although earlier this year remdesivir was used under EUA, on 22^nd^ October 2020 after three randomised controlled open labelled multi center clinical trial the antiviral drug (Veklury) Remdesivir from Gilead Science Incorporated has recently been approved by the USFDA as the first drug to get official approval for treatment of COVID-19 patients of 12 years and older of age with weighing at least 40 kg. They added to the knowledge that the drug achieved clinical recovery by five days faster and reduced the mortality rate of patients on ventilation support. They were instructed to be administered only in a hospital or healthcare setting comparable to inpatient hospital care. There must be benefit risk assessment to be followed to monitor the possible side effects like increased liver enzymes, allergic reactions, and nausea.^36^

Azithromycin - another agent which was tried, is a macrolide antibacterial, which has immunomodulating properties and decrease the exacerbations of cytokine production that normally occur during pulmonary respiratory disorders, reduce the chemotaxis of neutrophils to the lungs by inhibiting cytokines, IL-8, inhibiting neutrophil apoptosis, and blocking the activation of nuclear transcription factors.^27^

Tocilizumab: There have been evidences stating the use of tocilizumab has been useful to certain extent. The body temperatures have reached to normal in a single day, the clinical symptoms have reduced, there has been better oxygen saturation capacities, the percentage of lymphocytes and CRP levels have comer to normal values. Anecdotal reports have described good outcomes with the IL-6 receptor inhibitor tocilizumab but there are no published clinical data supporting its use. It is an IL-6 pathway inhibitor which is a monoclonal antibody, that acts by inhibiting the proinflammatory cytokines, T-cell activation, immunoglobulin secretion induction, haematopoietic precursor cell proliferation and differentiation stimulation. As the infection primarily causes chest tightness, shortness of breath, respiratory failure, the chest CT-shows opacities which was eventually decreased to a far extend by tocilizumab. This agent as well as sarilumab and siltuximab which also target the IL-6 pathway are being evaluated in clinical trial.^37^

Interferon beta 1-a: It has been under trials for its antiviral activity against SARS-CoV-2. The interferon therapy produces a cytopathic effect by reducing the infection and viral replication of coronavirus. The pre-treatment of interferon beta-1 b shows significant protection of post infection for 24-48 hours.^38^ There were notable difference in the discharge rates when patients received interferon beta 1-a in the early phase of disease with minor side effects like neuropsychiatric problems, agitation, mood swings, mild depression, hypersensitivity reactions, injection related problems (fever, chills, myalgia, headache) that were eventually tolerable and resolved during follow-ups.^39^ The recent studies demonstrates controversy in the use of interferon-I as treatment option in COVID-19 patients due to their activation of innate immune responses that promotes inflammatory cytokines production therefore the early use of interferon alpha decreased the mortality rates. There is future trial ongoing on IFN-III that exhibits antiviral activity without inflammatory efforts.^40^

Convalescent plasma: A new drug application is the use of convalescent plasma for patients with life threatening COVID-19 infections has been undertaken by the United States. Convalescent plasma is collected from persons who have retrieved from COVID-19 infections that may contain antibodies to SARS-CoV-2. This is a type of passive antibody therapy that involves the administration of antibodies against a foreign agent present in the body of a susceptible individual for the purpose of preventing the infectious disease due to that agent. This is a means of providing immunity at the earliest to the susceptible persons.^38^ The mechanism of action undertaken here is viral neutralisation. The sources of antibody for SARS-CoV-2 are human coalescent sera of recovered people, monoclonal antibodies, or preparations made in some animal hosts or engineered products that eventually produce human antibody. This procedure becomes a success only when there is an increase in potential donors quantitative. This treatment procedure is effective when used prophylactically rather than for treatment of disease. They neutralise the initial inoculums which is easier in the initial stages, they also work by modifying the inflammatory responses which is also achieved during initial stages. After administration, the antibody circulates in blood, reaches tissues, and provide protection against diseases. There are also risks associated with passive administration of convalescent sera including TRALI (transfusion related acute lung injury) and ADE (antibody dependent enhancement) of infection, and chances of re-infections. Current data suggest the use of convalescent sera is safe in patients having a high chance of mortality, elderly and vulnerable groups where benefits outweighs risk. Convalescent sera can be used as a precautionary for members of the same family where caring for COVID-19 patients at home is required. It would be an expedient measure in the middle of current chaos.^41^

Favipiravir: It is an RNA polymerase inhibitor that is available in some Asian countries for treatment of influenza and is being evaluated in clinical trials for treatment of COVID -19, but the published clinical data is pending. Favipiravir is a new type of RNA-dependent RNA polymerase (RdRp) inhibitor, they block the replication of viral genome.^12^

Arbidol is orally administered at a dose of 200 mg 3 times/day. The duration of treatment is no longer than 10 days. Arbidol is an antiviral used to treat influenza virus. A study has revealed that Arbidol can effectively inhibit novel coronavirus infection in about a concentration of 10-30 μM in vitro.^12^

Corticosteroids: The effect of early usage of corticosteroid treatment for SARS-CoV-2 is tried in treating immune-mediated pulmonary damage. There are clinical trials on hydrocortisone as well as methyl prednisone verses placebo are being checked for efficacy in reducing fever and decreasing the lung opacities are under trials. There are limitations to the therapy due to delayed viral clearances and immunosuppressive effects.^42^ RECOVERY trials performed at UK suggests corticosteroids like dexamethasone and methylprednisone has a good bioavailability at lungs and works only at the early stages of infection. Their anti-inflammatory properties help to reduce the systemic inflammation and excessive fluid in lungs and further alveolar damage. The one major problem associated with steroid therapy is the inhibitory effect on immune response after long term use leads to decreased viral clearance.^43^ Corticosteroids are beneficial in reducing hospital stay in critically ill mechanically ventilated COVID-19 patients when compared to non-ventilated patients.^44^

Niclosamide - an anticestodal drug functions by inhibiting oxidative phosphorylation and stimulating adenosine triphosphate activity in mitochondria, it also has broad antiviral activities and acts in combating COVID-19. Niclosamide suppresses the cytopathic effect of SARS-CoV at a concentration as low as 1 µM and inhibited SARS-CoV replication with EC50 value of less than 0.1 µM.^19^

## Repurposing of antiviral drugs for COVID-19 infections- considerations and limitations

There is a steeping increase in cases from day to day therefore the race to find appropriate treatment has accelerated a big challenge. Due to the urgent need even though some repurposed drugs gained attention at beginning due to the positive results from small scale studies the long term use shows there are drawbacks for every treatment.^45^ Drug repurposing is an attractive and readily available technique that uses the existing drugs for treating the unexpected sudden occurred challenging diseases thereby reduces the cost and timeline spent. On March 28^th^ 2020 the FDA granted EUA for HCQ and Remdesivir on May 1, 2020 but however on 15th June FDA revoked the EUA of HCQ. Several drugs like remdesivir a monophosphate prodrug used for potential treatment of Ebola virus was approved by FDA under emergency situations in severely ill COVID-19 patients, mefuparib (CVL218) a poly (ADPribose) polymerase1 inhibitor shows antiviral activity against SARS-COV-2, toremifene selective estrogen receptor modulator l used in breast cancer also had the ability to block the interactions of virus in MERS, SARS and inhibits the non-structural proteins of SARS-COV-2. Even the host targeting strategies of baricitinib, melatonin and dexamethasone approved for various inflammatory and autoimmune conditions paved the way for reduction of mortality in COVID-19 patients. There are also combination drug therapies for boosting the host immune responses but are presently under theoretical analysis and not tested in preclinical trials. Glucocorticoids (dexamethasone) have proven to be effective in mechanic ventilation hospitalised patients by increasing the viral clearance and reducing the mortality rates of severely ill COVID-19 patients. Androgen receptor inhibitors like spironolactone, eplerenone are having extensive safety profile against hypertension and inhibits aldosterone actions, they also provide possible antiviral and anti-inflammatory actions that reduce lung injuries. Similarly, the 5 alpha reductase inhibitors –dutasteride and finasteride, inhibit the TMPRSS2 expression. Statins have anti-inflammatory, immunomodulatory, antioxidative, antiarrhythmic effects therefore reduce the severity of ARDS and lung injury. Vitamin D with calcium metabolites provides immunological protection from COVID-19 viral activity and neutralize effects on TMPRSS2. N-acetyl cysteine inhibits inflammatory pathways, improves T cells, and reduces lung injuries and prevent the pairing of ACE2 with SARS-COV-2 virus.^46^

SARS–CoV-2 enters lung alveoli by receptor mediated endocytosis, via angiotensin converting enzyme 2 receptors. Therefore drugs inhibiting the AP2-associated protein kinase 2 can prevent the viral entry to certain extend. Baricitinib is an AP2-associated protein kinase2 and JAK inhibitor is used to prevent viral replication. Further the drug leronlimab a humanised monoclonal antibody (chemokine receptor type 5) antagonist and a nucleoside RNA polymerase inhibitor Galidesivir shows better survival rates and is potentially tested for further efficacy.^6^ A joint research team of the Shanghai Institute of Material Medica and Shanghai Tech University, China performed drug screening in silicon and an enzyme activity test, and they reported 30 agents with potential antiviral activity against SARS-CoV-2 on January 25, 2020. The same study was done in Chinese herbal medicines to extract the active ingredient against SARS-CoV-2.^12^ There are temporary evidence on the drug Ivermectin which can inhibit the replication of SARS-CoV-2 virus. Ivermectin is an FDA approved anti parasitic agent which is presently showing a high reduction in the infection caused COVID-19 RNA virus within 48 hours and with no toxicity levels. Supplementary clinical trial on run may potentiate its use as an antiviral agent.^47^

The challenges faced in drug repurposing are the difference in host environment of humans and cellular animal assays, lack of powerful clinical endpoints, few reproducible preclinical animal models, differences in sensitivity analysis, large heterogeneous populations, comorbid conditions variability, animal models with poor predictive efficacy, lack of monitoring parameters.^48^ Even the use of interferons combined with lopinavir/ritonavir in severe COVID-19 cases caused worsening of tissue damage,^45^ CQ showing macular retinopathy and cardiomyopathy, favipiravir is teratogenic thus pregnancy confirmed or suspected woman must avoid its use.^45^ The optimum dosing schedule with appropriate monitoring of the repurposed drugs are important in order to have an efficient target concentration as the drugs were implemented for different treatment paradigms.^33^ The Table 1 shows various drugs and its mechanism of action that repurposed for COVID-19 management.

**Table 1:** T mechanism of various drug therapies and reasons for the repurposing of the drug^49-66^

**Drug name**	**Mode of action**	**Drug target on SARS-CoV-2**	**Factors for the repurposing of the drug(indications)**	**Plausible adverse effects** ^61-66^	**Reference** ^49-60^
Chloroquine/hydroxychloroquine	They create an acidic endosomal pH that inhibits viral fusion with host cell receptors.	Interrupts the endosomal pathway and prevents viral entry and fusion with the host ACE2 receptors.	Antimalarial agent also used for the treatment of autoimmune diseases like rheumatoid arthritis. Their immunomodulatory, anti-inflammatory and anti-viral *in vitro* activities may have a role in viral pneumonia-like symptoms treatment of COVID-19.	Cardiomyopathy, arrhythmias, macular retinopathy	^49,50^
Darunavir/Atazanavir/Cobicistat	Inhibits viral protease	Viral proteins	Anti-HIV agents used in SARS-CoV infections.	Gastrointestinal disorders, hypersensitivity reactions	^49^
Lopinavir/Ritonavir	Viral protease inhibitor	Viral proteins	They are found effective in SARS and MERS-CoV. The combination inhibits viral replication and cleaves new virions in HIV.	Gastrointestinal disorders, glucose intolerance, hyperlipidemia, hepatotoxicity, icterus, retinal toxicity	^50^
Tocilizumab	Inhibits interleukin and cytokine proliferation	IL-6	Monoclonal antibodies effective in rheumatoid arthritis, cytokine release storm.	Hypersensitivity reactions	^49^
Remdesivir	Nucleotide inhibitors	RNA-dependent RNA-polymerase	Studies showed potential for SARS-CoV, MERS-CoV and Ebola viruses.	Multiple organ dysfunction syndrome, septic shock, hypotension and acute kidney injury	^51,52^
Umifenovir	Inhibits membrane fusion	Viral and host cellular membranes	Approved for influenza, HIV. Currently in phase IV trials for pneumonia associated COVID-19 infections.	Gastrointestinal adverse effects, raised transaminase levels, weight loss, hair loss	^49,50^
Favipiravir	Inhibits RNA polymerase	RNA dependent RNA polymerase	Studies show antiviral activity against the Ebolavirus, Lassa fever, and influenza. Trials conducted on coronavirus associated pneumonia-like fever	Teratogenicity	^49,50^
Camostat mesylate	They prevent the viral entry and fusion with TMPRSS2 proteins on host cell surfaces.	TMPRSS2 proteins. (protease inhibitors)	They regulate the cytokine expressions and inflammatory responses in chronic pancreatic fibrosis. *In vitro* studies show effective blockage of SARS-CoV-2 entry.	No reported adverse effects, monitor for common events like elevated peripheral blood eosinophilia, rash	^49,53^
Teicoplanin	Inhibits viral entry	Viral S proteins	Efficient against Ebola virus, HIV, MERS-CoV, and SARS-CoV infections.	Common adverse effect are rashes, ototoxicity	^54,55^
Oseltamivir	Inhibits viral replication	Viral polymerase and protease inhibitor	Antiviral agents used in HIV and HCV treatments.	Neuropsychiatric adverse events	^55^
Statins	Immunomodulatory effects, anti-inflammatory, and lipid-lowering agents	Inflammatory symptoms	They are mostly employed in patients with comorbid conditions. They reduce the host cell lung injury caused by COVID 19 infections.	Muscle related symptoms.	^55^
Angiotensin receptor blockers + zinc supplements	Reduce the viral multiplication and spread	ACE-2 receptors	Animal studies in mice show improved survival rates and reduce flu-like symptoms.	No reportable effects, commonly include headache, vomiting, diarrhoea	^45^
Sofosbuvir	Inhibits viral polymerase	RNA dependent RNA polymerase	Clinically effective against anti-HCV, Zika viruses	Pulmonary arterial hypertension	^56^
Ribavirin	They are nucleotide inhibitors.	Inhibits viral RNA-dependent RNA-polymerase (RdRp) enzymes	*In vitro* studies show antiviral activity against viral RdRp	Haemolytic anemia, teratogenic	^56^
Ribavirin+ Interferon α2b	Nucleotide inhibitor+ immune-modulators	Viral genome	This combination therapy shows effectiveness in RSV, HCV, and SARS-CoV. They possess antiviral and immunomodulatory effects that can be repurposed.	Neuropsychiatric effects	^57^
Telbivudine	Inhibits viral polymerase	Viral protein-M^pro^	Nucleoside analogues effective in hepatitis B virus	No reported effects, commonly include headache, liver problems, allergic reactions	^57^
Chlorpromazine	Inhibits viral fusion	Spike proteins, inhibition of clathrin-mediated endocytosis	Dopamine and adrenergic antagonists used against SARS, MERS and Ebola entry	No reported data, monitor for hypersensitivity, risk of glaucoma, urinary retention, agranulocytosis, allergy -NCT04366739	^58^
Nitazoxanide	Inhibits viral entry	Haemagglutinin (HA) expressions	Useful as anti-helminthic, production of interferons and inhibits in vitro MERS-CoV infections	Pruritis, hair loss, allergic skin reactions	^59^
Ivermectin	Inhibits nuclear transport of viral and host proteins.	IMPα/β1 transporter of RNA viruses.	The antiparasitic agent is effective against the Zika virus, pseudorabies virus.	Neurological adverse events	^60^

Vaccines: The Bharat Biotech, the countries pandemic vaccine leaders are collaborating with virologist at the university of Wisconsin–Madison and the vaccine company FluGen, for the project of developing vaccine against corona. They are in a process of developing an intranasal vaccine for COVID-19 called CoroFlu. CoroFlu is in the animal testing phase in the US, and is expected to move to human trials in the next three months. The intranasal route is selected as flu, RSV and corona enter through the naso-region, and hence produce mucosal immunity when given by nasal drop (just one drop is easy to be injected to the nasal route). Bharat biotech has commercialised 16 vaccines including the one developed against the H1N1 flu that caused the pandemic in 2009. Kawaoka lab will insert gene sequence from SARS-CoV-2 which cause COVID-19 into M2SR (which is a self- limiting version that induces immunity response against the flu). So that the new vaccine will induce immunity against the coronavirus.^67^ Codagenix (CDX-005) from Serum Institute of India manufactures intranasal live attenuated vaccine candidate for SARS-CoV-2 which are expected to be more efficient and patient friendly. As the virus mechanism remains uncertain, it is extremely critical to develop first generation vaccine within short period of time. Various vaccine developed and under development for COVID-19 across globe is summarized in Table 2. As prevention is better than cure the immunization programmes is far better for the future perspective. At present there are challenges developing a successful vaccine, but the future may be beneficial enough to tackle the changing strains of coronaviruses. As a matter of moment scientists’ tie-up to produce appropriate immune response to terminate the pandemic. There are several concerns, related to the vaccine development which includes reduced small sample size studies, estimation of safety profiles for elderly or minorities and vulnerable groups. Thus, it is difficult to evaluate the efficacy or generalise results as current trials have large gaps in the types of people being enrolled in phase III trials of vaccine development in different countries. It is therefore important to have large scale surveillance trials so that vaccine provides long term safety and prevents infection. Resistance to antivirals are the new emerging challenge in the raising pandemic. The regulatory authority of each country may provide EUA of vaccines or drugs before the formal approval to just tackle the spread of infection for the moment and monitor their utilization in severely ill patients.^68^ Despite of the burst of coronavirus being sudden there are many pharmaceutical companies trying hard for the isolation, identification, and management of the COVID-19 and it might take another 10 years for composing an effective vaccine.^69^

**Table 2:** Vaccines developed for SARS-CoV-2 infections^70-76^

**Developer**	**Vaccine**	**Type of vaccine**	**Phase 3 of clinical trial** ^70-73^	**Future outcomes** ^74-76^
Moderna - National Institute of Health	mRNA-1273	Genetic vaccine (lipid nanoparticle encapsulated-mRNA based vaccine) mRNA-1273 against SARS-CoV-2 targets the S spike proteins	Began on July 27^th^ 2020 –NCT04470427	Scientists all around the world are in high-pressure to find a potential treatment and vaccine to crack down the COVID -19 pandemic. Vaccines have the capacity to train the immune system to recognize and attack the virus when they encounter the host cells. Vaccines provide permanent cure and rapid targeted drug delivery. The major factors considered are the strength of antigens, vaccine delivery system and route of vaccination. The production of adequate amount of neutralising antibodies and periods of long term protection by vaccines is necessary to block viral pathogens. There can be adverse effects even in case of vaccine administration hence proper clinical phase trials with small animals and nonhuman primates are mandatory prior to human trials. The use of adjuvants can intercept the side effects to an utmost level. Of this intranasal route stimulates both cellular and humoral immunity and promises a higher level of protection so far in clinical studies. However the safety and efficacy profiles are crucial factors that determines the success of an ideal vaccine development.
CanSino Biologics Inc/Beijing Institute of Biotechnology (approved for limited use in China)	Ad5-nCoV	Recombinant viral vectored vaccine Ad type 5/non replicating viral vaccine	Began on September 15^th^ 2020 -NCT04526990	
Gamaleya Research Institute of Epidemiology and Microbiology (approval for early use in Russia)	Gam-Covid-Vac (Ad5+Ad26)	Viral vectored vaccine Adeno based Gam-COVID-Vac/non replicating viral vector	Began on September 28th 2020-NCT04564716
Johnson & Johnson (Beth Israel Deaconess Medical Centre in Boston)	Vaccines made with (Ad26.COV2.S) AdVac^R^ technology	Viral vectored vaccine-Ad26(ENSEMBLE trial)	Began on September7th 2020-NCT04505722
Novavax/Emergent Biosolutions	NVX-CoV2373	Protein based vaccine (VLP recombinant sub unit, full length S trimer/nanoparticle +matrix X (S-glycoprotein helps in binding to ACE-2 receptor and generate antibody against epitopes	Began on September 28th 2020-NCT04583995
Sinopharm-Wuhan Institute of Biological Products (approved for limited use in UAE)	Covid-19 vaccine	Inactivated/live attenuated coronavirus vaccine (after passage/inactivation virus lose virulence)	Began on July 2020-
Sinovac Biotech (approval for limited use in China)	CoronaVac/Picovac	Inactivated/live attenuated coronavirus vaccine (after passage/inactivation virus lose virulence	Began on July 21^st^ 2020-NCT04456595
Bharat Biotech (Indian Council of Medical Research + National Institute of Virology)	Covaxin (BBV152)	Inactivated/live attenuated coronavirus vaccine (after passage /inactivation virus lose virulence)	Began on October3rd 2020-
Laboratorio Elea Phoenix S.A. (Beijing institute of biological products)	Covid-19 vaccine	Inactivated SARS-CoV-2 vaccine	Began on September16th 2020-NCT04560881

## Conclusion

There have been no effective antiviral therapies or vaccines approved so far for SARS-CoV-2. Vaccines for pandemic infections require rapid development and high production capacity. Many of the vaccine candidates are under developmental phase with a good number in clinical trial phase. An appropriate therapy requires the identification of surface structure of spike glycoproteins for the development of antiviral drug that still has multiple challenges. Clinical trials have been initiated in different countries to develop effective treatment options, but this may take several years to trace out a reliable treatment therapy for patients. Larger sample size studies are needed to investigate the effects of antiviral therapies for COVID-19 infections leading to a better endpoint. Currently many clinical studies have been reported worldwide and several drugs are repurposed to tackle the new health emergency of COVID-19. But there are no sufficient data to suggest the treatment eradication of COVID-19 as there is lack in comparative studies. Wide population of patients involved RCT opens the era for evaluating and reassuring the efficacy of drug repurposed for COVID-19. It is early to predict the safety and efficacy of drugs repurposed as they might have an alarming side effect in the future trials. As there is no pre-existing immunity against the new virus the exploration of intranasal delivery techniques with mucosal immunity may be an eye opener. It is also important to acknowledge that there are so far no proven data supporting the standard use of any of these agents for COVID-19.

## Ethical Issues

There are no ethical issues.

## Conflict of Interest

Authors declare no conflict of interest in this study.

## Acknowledgements

We thank Dr. Shantikumar Nair, Dean of Research, Amrita Institute of Medical Science & Research Centre, and Dr. Sabitha M, Principal, Amrita School of Pharmacy for the facilities provided.
